# Effect of space allowance and mixing on growth performance and body lesions of grower-finisher pigs in pens with a single wet-dry feeder

**DOI:** 10.1186/s40813-020-00187-7

**Published:** 2021-01-06

**Authors:** Jordi Camp Montoro, Laura Ann Boyle, David Solà-Oriol, Ramon Muns, Josep Gasa, Edgar Garcia Manzanilla

**Affiliations:** 1 Pig Development Department, Animal and Grassland Research and Innovation Centre, Teagasc, Moorepark, Fermoy, Co. Cork P61 C996 Ireland; 2 Department of Animal and Food Sciences, Animal Nutrition and Welfare Service, Universitat Autònoma de Barcelona, 08193 Bellaterra, Spain; 3 Agri-Food and Biosciences Institute, Large Park, Hillsborough, Co Down, Northern Ireland BT 26 6DR UK; 4 UCD Veterinary Sciences Centre, University College Dublin, Belfield, Dublin 4, D04 V1W8 Ireland

**Keywords:** Animal welfare, Group size, Pig, Regrouping, Stocking density, Swine

## Abstract

**Background:**

Low space allowance (SA) and mixing may result in reduced growth performance (GP) and animal welfare issues because of adverse social behaviours directed to pen mates. This could be exacerbated in pens with single space feeders owing to social facilitation of feeding behaviour. The present study aimed to investigate the effect of SA and mixing on GP and body lesions (BL) in pens with one single space wet-dry feeder.

**Results:**

Two experiments were conducted on grower-finisher pigs from 10 to 21 weeks of age. In Exp1, pigs (*N* = 216) were assigned to three SA; 0.96 m^2^/pig (*n* = 6 pens; 10 pigs/pen; SA96), 0.84 m^2^/pig (*n* = 6; 12 pigs/pen; SA84) and 0.72 m^2^/pig (*n* = 6; 14 pigs/pen; SA72), in a randomized design. In Exp2, pigs (*N* = 230) were used in a 2 × 2 factorial randomized design considering SA and mixing as treatments. Pigs were assigned to two SA; 0.96 m^2^/pig (*n* = 10 pens; 10 pigs/pen; SA96) and 0.78 m^2^/pig (*n* = 10; 13 pigs/pen; SA78) and were either mixed or not at the entry to the finishing facility. GP was not affected by SA (*P* > 0.05) in either experiment. In Exp2, non-mixed pigs were 5.4 kg heavier (*P* <  0.001), gained 74 g more per day (*P* = 0.004), consumed 101.8 g more of feed per day (*P* = 0.007) and tended to have higher feed efficiency (*P* = 0.079) than mixed pigs from 11 to 21 weeks of age. Number of BL was affected by SA in both experiments. In Exp1, SA72 pigs had 74.4 and 97.4% more BL than SA96 and SA84 pigs at 20 weeks of age respectively (*P* <  0.01). In Exp2, SA78 pigs had 48.6, 43.6 and 101.3% more BL than SA96 pigs at 12, 16 and 21 weeks of age respectively (*P* <  0.05). Mixing did not affect the number of BL from 12 to 21 weeks of age in Exp2 (*P* > 0.05).

**Conclusion:**

Mixing had a considerable effect on growth performance thus, strategies to avoid or mitigate mixing should be considered. Although space allowance had no effect on growth performance, high number of body lesions in the lower space allowance indicates that space allowances equal or below 0.78 m^2^/pig are detrimental to the welfare of pigs despite following the EU legislation.

## Background

One of the greatest challenges for pig producers is to maximise pig production efficiency and minimise housing cost, without compromising animal welfare and farm sustainability. Housing costs are the second highest cost during the grower-finisher period after feed cost [1]. A higher number of pigs per pen, reduces the housing cost per pig as pens are used more efficiently [2]. However, for a given pen size, increasing the number of pigs per pen causes a reduction in the space allowance (SA), and either a reduction in feeder space per animal in long trough systems or an increase in the ratio of animals per feeder in facilities with an ad libitum feeding arrangement [3]. This may result in a reduction in productive performance [4–6]. Changing the number of pigs per pen may induce confounding on whether productive performance is affected by SA, feeder space or group size [7], although this method is the most common applied in commercial conditions. Nevertheless, recent research pointed out no effect of group size and feeder space on productive performance [1, 8, 9].

Kyriazakis and Whittemore [10] reported a formula where SA in m^2^ is expressed as: SA = *k* × BW^0.667^ where *k* represents a space allowance coefficient and BW^0.667^ represents the geometric conversion of body weight (BW) in kg to area. Gonyou et al. [11] reported that below 0.0336 m^2^/BW^0.667^ productive performance is affected. However, these thresholds are likely to change for each type of building, floor type, feeders, environmental enrichment, sex and pig’s genetics [9, 12], and animal welfare may be compromised before performance is affected. Space allowance is part of the Welfare Quality assessment of pig production [13] and it is well known that insufficient SA can lead to adverse social behaviours directed to pen mates, resulting in skin lesions, lameness, and tail biting [14]. These lesions are more sensitive indicators of pig welfare [15] than growth performance. Existing research recognises the critical role played by space allowance [7, 14, 16] and mixing [17] on the number of body lesions per pig as an indicator of poor animal welfare. The physical damage induced by aggression may end affecting pig performance causing carcass condemnations and economic losses for pig producers [18]. Moreover, damaging behaviour may contribute to chronic stress which affects both mental and physiologic natural state of the animals [19], thereby having detrimental implications to the efficiency and sustainability of swine production systems [20].

Mixing is a common strategy used in pig production to sort pigs by weight to reduce variability and facilitate management in the grower-finisher stage, even though it has minimal impact on the final variability in individual pig weights within a pen [7, 21]. In fact, there are indications that mixing affects productive performance by reducing average daily gain (ADG) and average daily feed intake (ADFI) [22, 23] and it also affects animal welfare as pigs show severe aggression after re-grouping in order to establish a new social hierarchy [17]. Mixing is unavoidable in facilities with large groups at the finisher stage [8, 24], or it may also depend on the previous management undertake during the farrowing and nursery period [25]. However, farrow-to-finish commercial farms with pens of 10 to 14 pigs per pen at the finishing stage, could facilitate the maintenance of intact litters from farrowing to slaughter with no mixing. Despite no references are available, such a penning arrangement with one single space wet-dry feeder is a common type of accommodation for grower-finisher pigs in Europe. Thus, studies optimizing this system for efficiency and animal health and welfare are needed.

Previous literature showed the effects of SA on growth performance in grower-finisher pigs [1, 4, 11] but the information on how mixing affects growth performance in grower-finisher pigs is scarce and little attention has been paid to whether space allowance and mixing interact with each other [22, 23]. Understanding how productive performance is affected by different space allowances and mixing in each system is important for pig producers, veterinarians and advisors to make better management decisions. In the present study we hypothesised that an interaction between mixing and SA exists and it affects pig productive performance and animal welfare. Therefore, the aim of the present study was to investigate the effect of space allowance and mixing on growth performance and body lesions, as a proxy for aggression, in pens with a single space wet-dry feeder during the grower-finisher stage.

## Methods

### Care and use of animals

Two experiments (Exp1 and Exp2) were conducted at the Teagasc Pig Research Facility in Fermoy, Co. Cork, Ireland. Both experiments received ethical approval from the Teagasc Animal Ethics Committee (TAEC 204/2018). Danish Duroc × (Large White × Landrace) grower-finisher pigs were housed in mixed sex pens with fully slatted concrete floor (2.4 × 4.2 m) containing a single wet-dry feeder (330 mm [Width] × 370 mm [Depth] × 1000 mm [Height]; MA37, Verba, Netherlands) and one supplementary nipple drinker. Water and pelleted feed were provided ad libitum. Temperature was controlled by a mechanical ventilation system with fan speed and air inlet area regulated by a climate controller. Pens were enriched with a larch wood post. Pigs were fed a single soybean meal-maize-wheat based finisher diet (16.2% Crude Protein, 9.7 MJ per Net Energy and 0.92% Standard Ileal Digestible Lysine per kg of feed) and remained in the facility until the first group of pigs reached 110 kg of BW and were sent to slaughter in both experiments.

In Exp1, a total of 216 pigs were used and moved as intact litter pens to the finisher accommodation at 10 weeks of age (26.3 ± 2.26 kg BW). Pigs were assigned per pen to three different SA; 0.96 m^2^/pig (10 pigs/pen; *n* = 6; SA96), 0.84 m^2^/pig (12 pigs/pen; *n* = 6; SA84) and 0.72 m^2^/pig (14 pigs/pen; *n* = 6; SA72), in a randomized design. Litter pens were adjusted to the SA treatments by removing pigs in case it was necessary. All SA were above the minimum space per pig set by European legislation based on live weight [26].

In Exp2, a total of 230 pigs were used in a 2 × 2 factorial randomized design considering SA and mixing as treatments. Pigs were moved to the finisher accommodation at 11 weeks of age (34.3 ± 3.25 kg BW). Pigs were assigned to two different SA; 0.96 m^2^/pig (*n* = 10 pens; 10 pigs/pen; SA96) and 0.78 m^2^/pig (*n* = 10 pens; 13 pigs/pen; SA78), all above the minimum space per pig set by European legislation based on live weight [26]. Mixing was applied randomly to 5 pens of each SA while the rest of the pens remained as litter pens. Litter pens were adjusted to the SA treatments by removing pigs in case it was necessary.

In both experiments, space allowances were adjusted by changing the number of pigs per pen as it would happen in field conditions. Space allowance coefficient (*k*) for each treatment were calculated using the formula SA = *k* × BW^0.667^ [10] and are reported in Table 1.

**Table 1:** Initial and final space allowance coefficient (*k*) for each treatment in experiment 1 and 2

	Experiment 1			Experiment 2	
Number of pigs/pen	10	12	14	10	13
Space allowance, m^2^/pig	0.96	0.84	0.72	0.96	0.78
Space allowance coefficient, *k* ^a^					
Initial ^b^	0.110	0.095	0.079	0.090	0.074
Final	0.042	0.037	0.031	0.042	0.034

^a^ The allometric expression of the space coefficient is as follows: *κ* = Space allowance (m^2^) / BW^0.667^ (kg)

^b^Initial body weight in experiment 1 was: 25.6 **±** 1.38 kg (0.96 m^2^/pig), 26.1 **±** 1.38 kg (0.84 m^2^/pig) and 27.4 **±** 1.38 kg (0.72 m^2^/pig). Initial body weight in experiment 2 was: 34.6 **±** 0.95 kg (0.96 m^2^/pig) and 34.1 **±** 0.95 kg (0.78 m^2^/pig)

### Measurements

#### Body weight, feed intake and feed efficiency traits

In both experiments, pigs were weighed per pen and BW was recorded every two weeks until the first group of pigs reached 110 kg of BW and were sent to slaughter. Average daily gain was calculated for every 2 weeks interval. Feed intake was recorded daily at a pen level, added for every 2 week period and ADFI was calculated. Feed conversion ratio (FCR) was calculated as $\frac{\mathrm{kg}\ \mathrm{of}\ \mathrm{feed}\ \mathrm{consumed}}{\mathrm{BW}\ \mathrm{gain}}$ for each 2-week period.

#### Pen efficiency

Overall pen efficiency was calculated for each treatment in both Exp1 and Exp2. Pen efficiency was calculated as $\frac{\mathrm{kg}\ \mathrm{daily}\ \mathrm{gain}}{\mathrm{sq}\ \mathrm{m}\ \mathrm{space}}$.

#### Body lesion counts

Following the Welfare Quality® criteria [13], the body of the pigs was divided into anterior, mid and posterior part. Body lesion was defined as either surface penetration of the epidermis or penetration of the muscle tissue [13]. Then, all skin lesions in each location were counted individually as body lesions and recorded on a check sheet [13]. In Exp1, body lesions were counted at 20 weeks of age before pigs started to go to slaughter. In Exp2, body lesions were counted at 12, 16 and 21 weeks of age.

### Statistical analyses

All data were analysed using SAS v9.4 (SAS Institute Inc., Cary, NC, USA). Each pen was considered as the experimental unit for all data analyses. In Exp1, the models included SA as fixed effects. In Exp2, models included SA, mixing and their interaction as fixed effects. Models for BW, ADG, ADFI, FCR and pen efficiency were analysed using general linear mixed model accounting for repeated measurements in both experiments. Initial BW was used as a covariable for BW, ADG, ADFI and FCR. Body lesions were analysed using a generalized linear mixed model in both experiments. Difference between treatment groups on body lesions were calculated as $\frac{\mathrm{Group}\ \mathrm{A}-\mathrm{Group}\ \mathrm{B}}{\mathrm{Group}\ \mathrm{B}}$. Multiple means comparisons were done using Tukey-Kramer’s correction in all cases. Alpha level for determination of significance was 0.05 and trends were identified as alpha of 0.10. Results for fixed effects are reported as least square means ± standard error mean.

## Results

### Body weight, feed intake and feed efficiency traits

Final BW, ADG, ADFI and FCR from 10 to 20 weeks of age, were not affected by SA in Exp1 (*P* > 0.05; Table 2); although SA96 pigs were numerically heavier and had lower FCR than SA84 and SA72 pigs by the end of the trial.

**Table 2:** Effect of space allowance on productive performance and body lesion counts in Exp1^1^

Trait	Space Allowance, m^2^/pig ^2^				*P*-value
	0.96	0.84	0.72	SEM	
BW, kg, 20 wk	103.5	100.6	99.8	1.38	0.162
ADG, g	1211.8	1155.0	1141.0	35.88	0.396
ADFI, g	2566.2	2558.9	2580.2	70.34	0.979
FCR	2.15	2.20	2.26	0.07	0.578
Body lesions ^3^, 20 wk					
Anterior	2.0 ^b^	2.3 ^b^	4.1 ^a^	0.40	< 0.001
Mid	1.0	1.0	1.6	0.26	0.263
Posterior	0.8 ^b^	1.1 ^ab^	1.7 ^a^	0.23	0.021
Total	3.8 ^b^	4.3 ^b^	7.5 ^a^	0.78	0.003

^1^ Body weight (BW), average daily gain (ADG), average daily feed intake (ADFI), feed conversion ratio (FCR) and body lesion counts from 216 grower-finisher pigs (6 pens/treatment; Least square means ± Standard error mean [SEM]) from 10 to 20 weeks of age, when the first group of pigs reached 110 kg of BW and were sent to slaughter

^2^ 0.96 m^2^/pig = 10 pigs/pen; 0.84 m^2^/pig = 12 pigs/pen; 0.72 m^2^/pig = 14 pigs/pen

^3^ Mean of the total number of body lesions counted at anterior, mid, posterior and total body regions on both sides of the body

^a,b^ Within rows, significant differences between groups (*P* <  0.05)

BW showed an interaction between SA and mixing in Exp2. SA78 non-mixed pigs were 6.1 and 6.5 kg heavier than SA96 and SA78 mixed pigs at 21 weeks of age (*P* <  0.001; Table 3). This interaction was not present for ADG, ADFI or FCR. Non-mixed pigs gained 74 g more per day (*P* = 0.004) and consumed 101.8 g more of feed per day (*P* = 0.007) than mixed pigs from 11 up to 21 weeks of age (Table 3). Non-mixed pigs tended to have lower FCR compared to mixed pigs from 11 to 21 weeks of age (*P* = 0.079; Table 3).

**Table 3:** Effect of space allowance × mixing on productive performance and body lesion counts in Exp2^1^

	Space Allowance ^2^							
	0.96 m^2^/pig		0.78 m^2^/pig			*P*-value		
Trait	Mixed	Non-Mixed	Mixed	Non-Mixed	SEM	Mixing	Space Allowance	Interaction
BW, kg, 21 wk	102.1 ^b^	106.4 ^ab^	101.7 ^b^	108.2 ^a^	0.95	< 0.001	0.472	< 0.001
ADG, g	983.0	1034.1	955.4	1052.3	21.82	0.004	0.836	0.309
ADFI, g	2150.3	2222.3	2125.6	2257.1	32.57	0.007	0.880	0.374
FCR	2.18	2.12	2.19	2.11	0.04	0.079	0.974	0.692
Body lesions ^3^, 12 wk								
Anterior	4.2	4.0	6.8	7.3	0.70	0.960	< 0.001	0.622
Mid	2.2	2.3	2.7	2.5	0.47	0.903	0.457	0.777
Posterior	1.0	0.7	1.1	1.1	0.28	0.590	0.371	0.505
Total	7.6	7.0	10.8	10.9	1.23	0.828	0.004	0.748
Body lesions, 16 wk								
Anterior	3.8	3.7	5.6	5.3	0.71	0.801	0.019	0.894
Mid	1.8	3.0	3.6	3.4	0.56	0.210	0.038	0.119
Posterior	2.2	1.9	2.9	2.8	0.36	0.600	0.021	0.641
Total	7.7	8.6	12.0	11.4	1.51	0.886	0.018	0.598
Body lesions, 21 wk								
Anterior	2.6	2.0	4.2	4.0	0.59	0.380	0.002	0.497
Mid	0.8	1.0	2.3	1.8	0.39	0.983	0.003	0.410
Posterior	0.6	1.0	2.0	1.8	0.36	0.569	0.001	0.316
Total	4.1	3.9	8.6	7.5	1.21	0.622	< 0.001	0.834

^1^ Body weight (BW), average daily gain (ADG), average daily feed intake (ADFI), feed conversion ratio (FCR) and body lesion counts (Least square means ± Standard error mean [SEM]) from 230 grower-finisher pigs in 20 pens grouped by space allowance × mixing from 11 up to 21 weeks of age, when the first group of pigs reached 110 kg of BW and were sent to slaughter

^2^ 0.96 m^2^/pig = 10 pigs/pen; 0.78 m^2^/pig = 13 pigs/pen

^3^ Mean of the total number of body lesions counted at anterior, mid, posterior and total body regions on both sides of the body

^a,b^ Within rows, significant differences between groups (*P* < 0.05)

### Pen efficiency

**Fig. 1:**
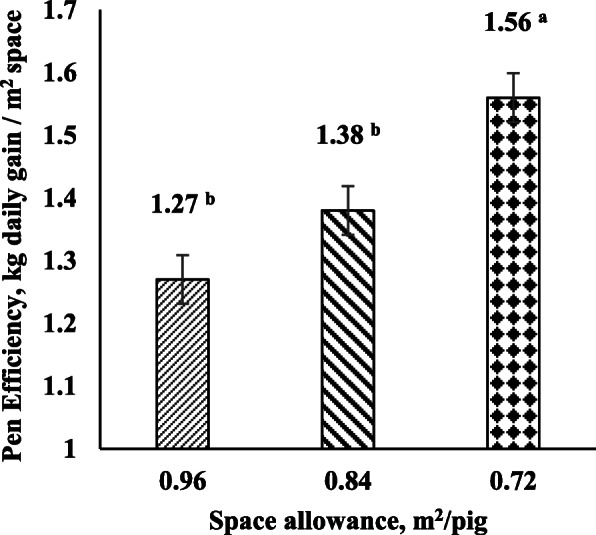
Pen efficiency of the space allowance treatments from 10 to 20 weeks of age in Exp1. ^a, b^ Significant differences between treatments (*P* < 0.05)

**Fig. 2:**
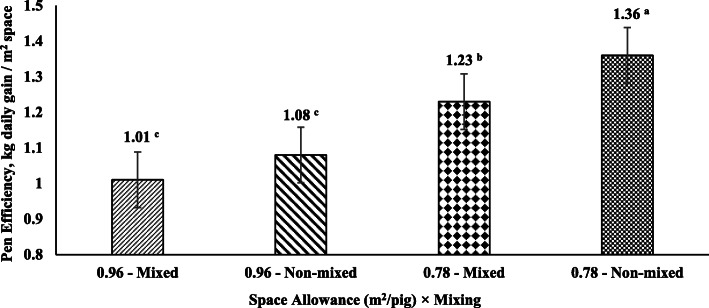
Pen efficiency of the space allowance × mixing treatments from 11 to 21 weeks of age in Exp2. ^a, b^ Significant differences between treatments (*P* < 0.05)

Pen efficiency increased by reducing SA during the grower-finisher period in both experiments (*P* < 0.001). SA72 pigs had higher overall pen efficiency compared to SA96 and SA84 pigs in Exp1 (*P* < 0.001; Fig. 1). Moreover, in Exp2, SA78 non-mixed pigs had higher overall pen efficiency than SA78 mixed pigs (*P* = 0.049) and SA96 non-mixed and mixed pigs (*P* < 0.001; Fig. 2). Also, SA78 mixed pigs had higher overall pen efficiency than SA96 non-mixed and mixed pigs (*P* < 0.05; Fig. 2).

### Body lesion counts

Body lesion counts were higher at the lower SA at 20 weeks of age in Exp1 (Table 2). SA72 pigs had 78.3 and 105.0% more lesions than SA84 and SA96 pigs on the anterior body region respectively (*P* < 0.001). There was no difference in counts on the mid body region between SA (*P* > 0.05). On the posterior body region, SA72 pigs had 112.5% more lesions than SA96 pigs (*P* = 0.021), although no differences were observed between SA84 pigs and the other groups (*P* > 0.05). In total, SA72 pigs had 74.4 and 97.4% more lesions than SA84 and SA96 pigs respectively (*P* < 0.01). In Exp2 (Table 3), body lesion counts were higher at lower SA, however no mixing and interaction effect were observed (*P* > 0.05). SA78 pigs had 72% more lesions on the anterior body region (*P* < 0.001) and 48.6% more lesions in total (*P* = 0.004) than SA96 pigs at 12 weeks of age. There was no difference in the mid and posterior body region between SA treatments at 12 weeks of age (*P* > 0.05). At 16 weeks of age, SA78 pigs had 45.3, 45.8, 39.0 and 43.6% more lesions than SA96 pigs on the anterior, mid, posterior and total body region respectively (*P* < 0.05). SA78 pigs had 78.3, 127.8 and 137.5% more lesions than SA96 pigs on the anterior, mid and posterior body region respectively at 21 weeks of age (*P* < 0.01). In total, SA78 pigs had 101.3% more lesions than SA96 pigs at the end of the trial (*P* < 0.001). Body lesion counts decreased from 16 to 21 weeks of age in all treatments (*P* < 0.05).

## Discussion

Small group pens with 10–14 grower-finisher pigs are convenient from a management point of view because they allow for a rapid monitoring of health and welfare issues in pigs, without the need to access the pen. This pen system is normally linked to wet-dry feeders as these optimize feed efficiency [27]. Despite no references are available, the authors’ experience in groups like EUPIG (https://www.eupig.eu/) or the ECPHM suggests that this is one of the most common systems in growing-finishing units in the EU. To understand how space allowance and mixing influence growth performance and welfare of pigs in this system, pigs were subjected to three space allowances (0.96, 0.84 and 0.72 m^2^/pig) in a first trial, and two space allowances (0.96 and 0.78 m^2^/pig) combined with mixing in the second trial. These space allowances were chosen above the 0.65 m^2^/pig minimum set by European legislation based on live weight [26] which is already criticised from a welfare point of view because of very low amount of shared space [28]. The space allowances were adjusted by changing the number of pigs per pen as it would be observed in current field situations. This fact could induce confounding on whether growth performance is affected by space allowance, feeder space or group size [7]. However, these factors are usually confused in any commercial conditions. Nevertheless, Schmolke et al. [8] observed no detrimental effect on growth performance of pigs housed at 10, 20, 40 and 80 pigs per pen with a space allowance of 0.76 m^2^/pig and one single wet-dry feeder provided for every 10 pigs. Moreover, Flohr et al. [1] also reported no effect of group size on ADG, ADFI and FCR in similar conditions to the present study. This results suggests that group size would not affect productive performance in the present study. Restricted feeder space could also impact growth performance [3]. Wastell et al. [9] compared group sizes of 20 and 26 pigs per pen and did not find any detrimental effect on growth performance with 10 pigs per wet-dry feeder space compared to 13 pigs per wet-dry feeder space considering a space allowance of 0.65 or 0.78 m^2^/pig. This results suggest that feeder space would not affect growth performance in the present study. Hence, the present study discusses space allowance as the main factor to affect growth performance regarding the pen system studied.

In terms of space allowance, previous studies observed that decreasing space allowance resulted in a poorer growth performance in pigs with space allowances of 0.65 m^2^/pig and similar slaughter weights [4, 5, 9] or 0.80 m^2^/pig when marketed to slaughter weights up to 138 kg of BW [29]. Overall, all these studies can be compared using the allometric approach expressing space allowance as a coefficient (*k*) [10]. Gonyou et al. [11] stated that the critical *k* value below which growth performance is affected as space allowance is further decreased, ranges from 0.0317 to 0.0348 over all data sets analysed using a broken-line analysis. In our study, pigs with 0.72 and 0.78 m^2^/pig reached the critical *k* value by the end of the trial when the first group of pigs reached the marketed weight (i.e. 110 kg of BW) and were sent to slaughter. Thus, the growth performance of the pigs would not be compromised during the grower-finisher period if space allowance is established based on the critical *k* value at the marketed weight.

Mixing affected growth performance during the whole grower-finisher period in Exp2. The drop in ADG and ADFI is consistent with previous literature which observed that mixed pigs had decreased ADG and ADFI when they were followed in a 4 week experiment at the beginning of the grower-finisher period [22, 23]. Stookey and Gonyou [30] also observed a depressed ADG in mixed pigs after being mixed for a 2 week period when they had 83 kg of BW. The present study showed that mixing causes a severe effect on growth performance in currently modern facilities and genetics, and strategies to avoid mixing or mitigate it are an important issue for future research [31, 32].

The underlying hypothesis in this study was that space allowance and mixing interact with each other in current field situations. The study found that mixing effect on final BW is exacerbated at lower space allowances (i.e. 0.78 m^2^/pig). However, this interaction did not show up in any of the other variables and should be checked for repeatability in further experiments. Hyun et al. [22] reported that when pigs are subjected to multiple concurrent environmental stressors such as high ambient temperature, regrouping and low space allowance, the final effect over productive performance is additive.

In terms of animal welfare, the current study found that the number of body lesions increased at lower space allowances. This finding is in accordance with previous literature which reported a strong relationship between space allowance and body lesions [7, 14, 16]. Anil et al. [7] stated that animal welfare is enhanced at higher space allowances in terms of postural behaviour, lower injuries and aggression. Space allowance affected the number of body lesions during the whole grower-finisher period. Nevertheless, the number of body lesions decreases as the pigs get heavier which is in accordance with previous studies [33]. A possible explanation for this might be related to the pigs’ experience and ability to adapt to their social environment and being in a stable group for a long time which benefits the long term welfare of the pigs [34, 35]. The present study raises the possibility that there is a threshold between 0.78 and 0.84 m^2^/pig which an increase in the number of body lesions due to space allowance is observed. Still, the number of body lesions as a proxy for aggression may vary because of other factors not controlled in the present study, and moreover, it could also be related to the pen design and the wet-dry feeder space per pig.

One interesting finding was that highest body lesion counts were seen in the anterior body region which is consistent with fighting for access to the feeder [36]. Single space wet-dry feeders may allow to accommodate a high number of pigs per feeder space without having a detrimental effect on growth performance [9, 37]. However, López-Vergé et al. [38] observed that pigs allotted to more feeder spaces had low body lesion counts and tended to have low BW variability within pen by the end of the grower-finisher period.

The results provided in the present study indicate that animal welfare may be compromised before growth performance is affected. Averós et al. [39] suggested a critical *k* value of 0.039 for lying behaviour with a broken-line analysis. This *k* value is higher than the 0.0336 reported by Gonyou et al. [11] below which productive performance is affected. High number of body lesions caused by competition or aggression, are likely associated with detrimental implications for pig health and performance due to immunosuppression caused by the social stress [40–42]. This fact may be exacerbated in farms that have more infectious diseases in comparison to the farm where the trial was performed, which is free of the main infectious diseases. Ultimately, compromised animal welfare has detrimental implications to the sustainability of the swine production system [20].

Mixing pigs leads to agonistic social behaviour mainly within the first 24 h [43]. However, the current study observed that the number of body lesions due to aggression in mixed groups is the same as the non-mixed groups after 1 week of being mixed. This finding is consistent with previous studies which may be explained by the establishment of the social hierarchy [44–46]. Nevertheless, mixed pigs showed a poor growth performance compared to their counterparts in the study. This could be related to the social network properties and chronic stress that are not shown in body lesions [19, 46, 47].

Regarding pen efficiency, this study supports evidence from previous observations [7] which showed that pigs in lower space allowances had higher overall pen efficiency. In addition, pen efficiency showed an interaction between space allowance and mixing which indicates that pen efficiency in lower space allowance may be affected when pigs are mixed. These results may encourage pig producers to seek optimal space allowances to optimize overall efficiency and reduce housing costs. Nevertheless, the present study also observed that pig welfare is aggravated in low space allowances. This and if other environmental stressors such as high ambient temperature, mixing or the farm sanitary status are considered, pig performance and pig producer’s income may be mitigated even with improved pen efficiency. Therefore, further studies considering economic analyses on how environmental stressors affect pig performance and welfare in different space allowances are needed.

## Conclusions

This study provides a deeper insight into how space allowance and mixing affect growth performance and animal welfare in pens of 10–14 grower-finisher pigs with one single wet-dry feeder. Mixing appears to have a considerable effect on growth performance although the number of body lesions is not affected once social hierarchy is established. Strategies to avoid or mitigate it are recommended in current field situations with a similar pen design. Space allowance will not compromise growth performance if it is established based on the critical *k* value at the marketed weight. Nevertheless, the increase on the number of body lesions in lower space allowances indicates that 0.72 and 0.78 m^2^/pig are detrimental to the welfare of pigs in single wet-dry feeder pens despite being compliant with the EU legislation. Animal welfare is affected before productive performance. Then, farmers should take it into account to maximise growth performance and overall efficiency of the facility without sacrifice animal welfare as a market concept.

## Data Availability

The datasets used and analysed during the current study are available from the corresponding author on reasonable request.

## Abbreviations

ADG: Average daily gain
ADFI: Average daily feed intake
BW: Body weight
Exp1: Experiment 1
Exp2: Experiment 2
FCR: Feed conversion ratio
SA: Space allowance
SA96: 0.96 m^2^/pig
SA84: 0.84 m^2^/pig
SA78: 0.78 m^2^/pig
SA72: 0.72 m^2^/pig
